# Clinical diagnoses vs. autopsy findings in early deceased septic patients in the intensive care: a retrospective cohort study

**DOI:** 10.1007/s00428-020-02984-5

**Published:** 2020-12-11

**Authors:** Rob G. H. Driessen, Bartholomeus G. H. Latten, Dennis C. J. J. Bergmans, Riquette P. M. G. Hulsewe, Johanna W. M. Holtkamp, Iwan C. C. van der Horst, Bela Kubat, Ronny M. Schnabel

**Affiliations:** 1 Department of Intensive Care Medicine, Maastricht University Medical Center+, Maastricht, The Netherlands; 2 Department of Cardiology, Maastricht University Medical Center+, Maastricht, The Netherlands; 3 Department of Pathology, Maastricht University Medical Center+, Maastricht, The Netherlands; 4 Department of Intensive Care Medicine, St. Jans Gasthuis, Weert, The Netherlands

**Keywords:** Autopsy, Intensive care, Sepsis, Septic shock, Diagnostic discrepancy

## Abstract

Early death in sepsis occurs frequently; however, specific causes are largely unknown. An autopsy can contribute to ascertain causes of death. The objective of the study was to determine discrepancies in clinical diagnosis and postmortem findings in septic intensive care unit (ICU) patients deceased within 48 h after ICU admission. All septic ICU patients who deceased within 48 h after ICU admission were identified and included. Four intensivists determined the clinical cause of death by medical record review. An autopsy was performed within 24 h of death. Clinical diagnosis and postmortem findings were compared and classified as autopsy-identified missed clinical diagnoses and autopsy-refuted diagnoses. Class I and II missed major diagnoses using the Goldman criteria were scored. Between 2012 and 2017, 1107 septic patients were admitted to ICU. Of these, 344 patients (31%) died, of which 97 patients (28%) deceased within 48 h. In 32 (33%) early deceased patients, an autopsy was agreed. There were 26 autopsy-identified missed clinical diagnoses found, mostly myocardial infarction (*n* = 4) and pneumonia (*n* = 4). In four patients (13%), a class I discrepancy was found. In fourteen patients (42%), a class II discrepancy was found. In conclusion, an autopsy is an important diagnostic tool that can identify definite causes of death. These diagnoses deviate from diagnoses established during admission in early deceased sepsis patients.

## Background

Sepsis is a life-threatening syndrome following a dysregulated host response to infection [1]. It still causes major public health concerns and has an increasing incidence [2]. Early death occurs in one-third of these patients [3]; however, studies investigating specific causes of death are scarce.

Autopsy, being the ultimate diagnostic test [4], is a reliable diagnostic tool in determining causes of death in critically ill patients. It also has an educational role, as studies show that attending necropsies foster clinical problem solving [5] and most students describe autopsies as educationally useful [6]. However, autopsy rates have been declining over the past few decades [7], possibly because of a lack of reimbursement, the fear of disclosing mistakes, and the conception that advances in medical technology diminish the additional value of autopsies [4].

Nevertheless, several studies have shown persisting discrepancies between clinical and pathological diagnoses in critically ill patients [8–11], reporting class I discrepancies in patients ranging between 3 and 16% of patients [12]. Class I discrepancies according to the Goldman classification are diagnostic errors that would have changed clinical management and possibly led to longer survival of patients. Class II discrepancies are errors that probably would not have changed therapy. Almost two-thirds of the class I discrepancies were found in patients with known infection [11]. Furthermore, identifying definite causes of (especially early) death in sepsis patients may improve clinical care and point out patient categories that might not benefit from specific interventions. To our knowledge, autopsy findings in septic patients dying within 48 h of ICU admission are sparse.

This study’s main objective is to determine the proportion of discrepancies in clinical diagnoses and postmortem findings in early deceased septic patients. Therefore, we conducted a retrospective cohort study, comparing clinical and autopsy diagnoses in patients with sepsis and septic shock who died within 48 h after ICU admission.

## Material and methods

### Setting

This study was conducted at the Maastricht University Medical Center+, a tertiary care, 715-bed university hospital in the Netherlands with 33 intensive care unit (ICU) beds with approximately 2500 annual admissions.

### Study population

Patients admitted to ICU are systematically screened for sepsis since 2012, and we entered all patients admitted with sepsis in ICU in a prospectively recorded database. All patients diagnosed with sepsis and deceased within 48 h after admission in ICU, between 2012 and 2017, were included in the study. Admission with sepsis was defined as any ICU admission clinically coded as infection and at least one organ dysfunction, according to the Surviving Sepsis Campaign guidelines of 2012 [13]. Septic shock was defined as sepsis with circulatory failure according to the Third International Consensus Definitions for Sepsis and Septic Shock (Sepsis-)3 criteria [1]. Families of eligible patients who died were routinely approached and requested permission to perform an autopsy on the deceased relative.

### Collection of data

For all early deceased septic patients, data on gender, age, source of infection, comorbidities (NYHA class IV cardiac failure, chronic restrictive or obstructive respiratory failure with functional impairment, hepatic cirrhosis, and immunosuppression), active malignancy, and APACHE score was recorded. Furthermore, the presence of acute renal failure (defined as creatinine above 175 mmol/L), coma on admission (defined as Glasgow coma scale ≤8 without sedation), and severe leucocytosis or leukopenia (> 40 or < 1/mm^3^) were also recorded.

### Clinical diagnosis

Clinical causes of death were investigated by manual analysis of the medical record by an expert panel of four independent physicians. This was based on information at ICU admission and during the length of ICU stay. Data collected included demographics, pre-existing medical conditions and severe comorbidity, presence of active malignancy, and source of infection. Clinical causes of death were based on judgement of the four intensivists as described, and in case of non-agreement between the intensivists, consensus could be reached in all cases.

### Pathological diagnosis

An autopsy was generally performed within 24 h of death. Before the autopsy, the pathologist was given relevant clinical information such as underlying diseases and clinical causes of death. This was performed by a standardized written request form for the autopsy, filled in by the treating physician. The pathologist was not blinded for the digital hospital record. A complete autopsy was performed by a pathology resident, supervised by a pathologist. The autopsy included macroscopic and microscopic examination, including histological analysis. The pathologists described their findings in a written report; both clinical and pathological diagnoses were made independently. The pathological diagnoses were determined by manually reviewing all the autopsy reports by the expert panel.

### Comparison of clinical and pathologic diagnoses

Clinical diagnoses and postmortem findings were compared, and discrepancies were classified into two categories: autopsy-identified missed clinical diagnoses and autopsy-refuted clinical diagnoses. The expert panel also classified class I and class II discrepancies using the Goldman criteria [14]. A class I missed major diagnosis being a diagnosis that would have changed patient management and possibly resulted in cure or prolonged survival. Class II missed diagnoses are defined as major discrepancies that probably would not have changed therapy in these patients, because patients were already receiving appropriate therapy, effective therapy was not available, or the patients were too sick to receive appropriate therapy. Discrepancies were classified based on consensus between all four intensivists.

### Statistics

Numerical variables were expressed as mean ± standard deviation (SD) and categorical variables as numbers and percentages. Means were compared with the *t* test for numerical variables and chi-square test for categorical variables. All statistical analyses were performed using SPSS version 22.0 (IBM, Armonk, NY).

## Results

### Study population

During the 5-year study period, from 2012 to 2017, 1107 patients were admitted with sepsis in ICU and 344 patients (31%) deceased in ICU. Ninety-seven patients deceased within 48 h after admission. An autopsy was performed in 32 (33%) of early deceased septic patients. The reason for not performing an autopsy was almost invariably refusal by the relatives.

Patient characteristics are shown in Table 1. Of the 97 early deceased patients, 62% were men, 56% were older than 65 years of age, and the mean APACHE II score was 32.7 ± 7. Patients often had severe comorbidity (58%), and underlying active malignancy was present in 51%. There were no statistically significant differences in demographic variables or comorbidity between the patients in the autopsy group and the non-autopsy group. The primary source of infection was the abdomen (37%), followed by the lung (31%). In 24% of patients, no definite source of infection could be determined. There were significantly more infections of unknown origin in the autopsy group compared to the non-autopsy group (36% vs. 15%, *p* = 0.021). On the other hand, there were more pulmonary infections in the non-autopsy group compared to the autopsy group (15% vs. 39%, *p* = 0.016).

**Table 1:** Patient characteristics

Variables	All patients	Autopsy	No autopsy	*p* value
	*N* = 97	*N* = 33	*N* = 64	
Demographics				
Male gender	61 (63%)	24 (73%)	37 (58%)	0.15
Age				0.30
≤ 44	2 (2%)	1 (3%)	1 (2%)	
45–54	9 (9%)	3 (9%)	6 (9%)	
55–64	22 (23%)	7 (22%)	15 (22%)	
65–74	33 (34%)	10 (30%)	23 (37%)	
≥ 75	31 (32%)	12 (36%)	19 (30%)	
Prognostic scoring				
APACHE II	32.7 ± 9	33.7 ± 7	32.2 ± 9	0.40
Severe comorbidity^a^	55 (57%)	16 (45%)	39 (63%)	0.24
Acute renal failure^b^	44 (45%)	18 (55%)	26 (41%)	0.64
Comatose at admission^c^	22 (23%)	7 (21%)	15 (23%)	0.80
Acidosis at admission pH < 7.25	80 (82%)	30 (91%)	51 (80%)	0.16
Leucocytes >40 or < 1/mm^3^	28 (29%)	10 (30%)	18 (28%)	0.82
Active malignancy	49 (51%)	20 (61%)	29 (45%)	0.15
Infection source				
Lung	30 (31%)	5 (15%)	25 (39%)	0.016
Abdominal	37 (38%)	15 (45%)	22 (34%)	0.29
Urinary tract	1 (1%)	0 (0%)	1 (2%)	
Cutaneous	4 (4%)	0 (0%)	4 (6%)	
Mediastinum	1 (1%)	0 (0%)	1 (2%)	
CNS	0 (0%)	0 (0%)	0 (0%)	
Catheter	0 (0%)	0 (0%)	0 (0%)	
Other	1 (1%)	1 (3%)	0 (0%)	
Unknown	23 (24%)	12 (36%)	10 (15%)	0.021
Clinical diagnosis				
Primary infection-related MOF^d^	36 (37%)			
Mesenteric ischemia/perforation	22 (23%)			
Cardiac arrest	21 (22%)			
End of life decision	13 (13%)			
Necrotizing fasciitis	4 (4%)			

^a^NYHA IV cardiac failure; chronic restrictive or obstructive respiratory failure with functional impairment, hepatic cirrhosis, immunosuppression

^b^Creatinine >175 mmol/L

^c^Glasgow coma scale ≤8 without sedation

ͩMultiple organ failure

### Clinical diagnosis

The main clinical cause of death in these early deceased septic patients was multiple organ failure related to the primary infection, occurring in 37% of patients. Furthermore, mesenteric ischemia was seen in 23% and death after unsuccessful cardiopulmonary resuscitation was seen in 22% of deceased patients. In Table 1, the clinical causes of death found by the expert panel are mentioned.

### Comparison of clinical diagnoses and autopsy findings

Table 2 shows the discrepancies between clinical diagnoses and autopsy findings in early deceased sepsis patients. We identified 26 (81%) autopsy-identified missed clinical diagnoses in 32 autopsies, most often myocardial infarction (*n* = 4) and pneumonia (*n* = 4). In 3 patients, the autopsy revealed a malignancy that was not clinically recognized (1 case of lung cancer, 1 case of hepatocellular carcinoma, and 1 case of gastric cancer). Three other patients had an unidentified haemorrhage, 2 located intra-abdominal and 1 intra-cerebral. Clinically undetected mesenteric ischemia was identified in two patients during the autopsy, and also refuted as clinical diagnosis in a patient. The same applied to pancreatitis.

**Table 2:** Major discrepancies between clinical diagnoses and autopsy findings, including class I errors (˟) (1 myocardial infarction, 1 ruptured aneurysm, 1 bleeding fistula, and 1 abdominal bleeding)

Diagnosis	Autopsy-identified missed clinical diagnosis	Autopsy-refuted clinical diagnosis
Myocardial infarction	4˟	
Pneumonia	4	1
Cancer	3	1
Haemorrhage	3˟	
Mesenteric ischemia	2	1
Cirrhosis	2	
Pancreatitis	2	1
Pulmonary embolism	1	1
Ruptured aortic aneurysm	1˟	
Renal infarction	1	
Fistula	1˟	
Intracardiac thrombus	1	
Intra-cerebral bleeding	1	
Total	26	5

According to the Goldman classification criteria, we identified class I errors in 4 patients (13%), meaning this would have changed management and possibly prolonged survival of these patients. This included 1 myocardial infarction, 1 ruptured thoracic aortic aneurysm, 1 bleeding fistula between the iliac artery and the small bowel, and 1 abdominal bleeding after percutaneous drainage of cholecystitis.

In 14 patients (42%), we found class II errors according to Goldman, meaning these were major discrepancies that would probably not have changed management because treatment was already given or not available, or patients were too sick to receive the appropriate treatment (for instance, surgery for bowel ischemia in a patient with deep septic shock). Class II errors included bowel ischemia (*n* = 4), myocardial ischemia (*n* = 4), and pneumonia (*n* = 3).

## Discussion

The present study aimed to determine the accuracy of clinical diagnoses and identify discrepancies with autopsy findings (especially class I discrepancies) in patients with sepsis and septic shock dying within 48 h after ICU admission. We observed an autopsy rate of 33% in this study population, and 26 autopsy-identified missed clinical diagnoses were found. In 13% of patients, a class I error was identified, meaning this would have changed management and possibly prolonged survival of these patients. In 42% of patients, a class II error was found.

To our knowledge, this is the first study investigating autopsy findings in this specific group of early deceased septic ICU patients. The autopsy rate of 33% is in range of earlier ICU autopsy studies [12] and much higher than the global autopsy rate in the Netherlands which is 2.7% [15]. The ICU studies report autopsy outcomes in general ICU populations, most often mixed medical and surgical patients. Most of these studies are also retrospective in origin, except for the prospective study by Combes et al. [10]. This study reported an autopsy rate of 53% in 315 deceased ICU patients and identified class I errors in 10% of patients.

Our study identified a class I error in 13% of patients, falling in the upper range of those reported in other ICU studies (3–16%). This discrepancy rate is in line with the findings of Silfvast et al., showing that 62% of class I diagnostic errors occur in patients with existing infections [11]. In a systematic review concerning ICU patients, and over 5000 autopsies, a class I error was reported in 8% [16]. Class I missed diagnoses are clinically the most important, as they would have changed treatment and possibly impacted survival. Despite improvements in technology, including imaging, these major discrepancies between clinical and autopsy diagnoses remain consistent over time at approximately 10% [14, 17].

We identified 26 missed clinical diagnoses, predominantly myocardial infarction, pneumonia, cancer, and haemorrhage. These categories of diagnoses are in line with other ICU autopsy studies [8, 10] and also with findings in the general (non-ICU) population [18]. We classified fourteen of these diagnoses as class II diagnoses, according to Goldman, meaning that they would probably not have changed therapy in these patients. Our study population represents very sick patients, often in deep septic shock, meaning that sometimes appropriate therapy for these diagnoses was not possible because of the severity of the shock. Although premortem diagnosis for some diseases (myocardial infarction and pulmonary embolism) is improved, infections, especially in immunocompromised patients, emerge as a more common cause of death and the anatomic location of infection is sometimes only detected by an autopsy [4]. In the present study, in 24% of deceased patients, the origin of infection was unknown, and, in the patients undergoing autopsy, this percentage (36%) was significantly higher.

Myocardial infarction was described in four septic patients undergoing autopsy in this study. Postmortem diagnosis of myocardial infarction can be complicated, especially in sepsis patients and the literature describes several diagnostic pitfalls [19]. In three of the four patients, a postmortem angiography was performed, showing three-vessel disease in two patients and one-vessel disease (RCA 75%) in one patient and no discrimination of a thrombus. In all four patients however, recent ischemia was demonstrated, in two patients by nitro blue tetrazolium (NBT) staining, and in all patients, this was confirmed with microscopic findings suggestive of ischemia (eosinophilic bands in the cytoplasm of cardiomyocytes). One patient underwent cardiopulmonary resuscitation (CPR) and this could explain the finding of myocardial ischemia at the autopsy. This underlines that unravelling the mechanism leading to myocardial ischemia in these patients can be challenging [19].

In four patients, pneumonia was found during autopsy. In three of these patients, a purulent effusion was found in the bronchi during the autopsy. Microscopic changes compatible with pneumonia like damaged alveolair septa and intra-alveolair presence of macrophages and neutrophilic granulocytes were found in all four patients. The fact that mesenteric ischemia is identified as both missed and refuted diagnosis with an autopsy points out that it is a challenging diagnosis, often occurring in patients with septic shock and multiple organ failure [20]. The same seems to be true for pancreatitis in these patients, especially since the pancreas undergoes rapid autolysis after death. It is difficult to distinguish premortem necrosis from autolysis [21], particularly when the inflammatory response is attenuated as can be the case in immunocompromised patients.

Studies have shown that as autopsy rate rises, the number of major misdiagnoses falls [22], pointing out the educational value of autopsy. In our center, the intensivist who took care of the deceased patient attends the final part of the autopsy and discusses the results with the pathologist. This facilitates the opportunity to look at the results in a clinical context and learn from each other. Indeed, previous studies demonstrate the importance of attending autopsies for deductive reasoning and awareness of the large proportion of patients with multiple diseases [23, 24].

Our study has several limitations. First, because we are investigating a specific study population (early deceased septic patients), the number of patients is small, limiting generalizability. An autopsy was performed in only 33% of patients, which might introduce bias; however, the autopsy group was largely comparable to the non-autopsy group. Furthermore, the study was performed in a retrospective manner. Nevertheless, given the paucity of data on this matter, our study provides valuable insights concerning the value of autopsy in sepsis patients dying early after admission.

In conclusion, despite technical improvements, in patients with sepsis who died early in the ICU, discrepancies between clinical diagnosis and postmortem findings are often identified. In 13% of patients, these discrepancies might have changed therapy or prolonged survival, underlining the importance of autopsies for patient care, understanding pathophysiology and epidemiology of sepsis patients.
